# Supplementary material for: Integrative Analysis of Transcriptomics and Proteomics for Screening Genes and Regulatory Networks Associated with Lambda-Cyhalothrin Resistance in the Plant Bug Lygus pratensis Linnaeus (Hemiptera: Miridae)
Source: Int J Mol Sci. 2025 Feb 18;26(4):1745. doi: 10.3390/ijms26041745 (PMC11855015; doi:10.3390/ijms26041745)
Supplement: Supplementary file 1 [file ijms-26-01745-s001.zip › ijms-3369969-supplementary.pdf]

Supplementary Table S1 Evaluation and statistics of sequencing data of *Lygus pratensis*

Sample	Raw Reads	Clean Reads	Before Filter Data(bp)	After Filter Data(bp)	GC(%)	Q20(%)	Q30(%)
S-1	61711438	60541304	9256715700	8946719473	47.69	98.75	96.18
S-2	43837472	43016610	6575620800	6357777705	47.16	98.77	96.24
S-3	49540598	48716882	7431089700	7206822241	44.92	98.86	96.49
R6-1	52240094	51280762	7836014100	7584417466	47.28	98.79	96.28
R6-2	55690058	54657688	8353508700	8079988451	46.50	98.8	96.33
R6-3	49829226	48932156	7474473900	7238488501	46.77	98.81	96.34
R14-1	55171046	54053926	8275656900	7993510258	46.54	98.78	96.25
R14-2	53918758	52946860	8087813700	7834552277	46.34	98.84	96.41
R14-3	60014384	58944718	9002157600	8730656865	46.43	98.84	96.41

Note: Sample: The sample identifier, used to distinguish different experimental groups or replicates. Raw Reads: The number of raw sequencing reads generated directly by the sequencing instrument. Clean Reads: The number of high-quality reads after quality control and filtering. Before Filter Data (bp): The amount of raw data before filtering (in base pairs). After Filter Data (bp): The amount of high-quality data after filtering (in base pairs). GC (%): The percentage of G and C bases in the sequencing data, reflecting the base composition. Q20(%): The percentage of bases with a quality value ≥ 20, indicating the accuracy of the sequencing data. Q30(%): The percentage of bases with a quality value ≥ 30, further reflecting the high-quality portion of the data.

Supplementary Table S2 Statistical table of assembly results

Gene No.	GC percentage	N50	Max length	Min length	Average length	Total assembled bases
82,919	42.5663	1655	27860	201	880	72,979,052

Note: Gene No.: The total number of assembled genes. GC percentage: The percentage of G and C bases in the assembled gene sequences. N50: An important metric for assembly quality, representing the length at which half of the bases are contained in sequences longer than or equal to this length. Max length: The length of the longest assembled gene sequence. Min length: The length of the shortest assembled gene sequence. Average length: The average length of the assembled gene sequences. Total assembled bases: The total number of base pairs assembled.

Supplementary Table S3 Annotated statistical table of four databases

Total unigenes	Nr	Swissport	KOG	KEGG	Annotation genes	Unknown gene
82,919	24,687	16,617	15,840	10,499	24,859	58,060

Note: Total unigenes: The total number of unigenes obtained. Nr: The number of genes annotated in the NCBI non-redundant (Nr) database. Swissport: The number of genes annotated in the Swiss-Prot database. KOG: The number of genes annotated in the Eukaryotic Orthologous Groups (KOG) database. KEGG: The number of genes annotated in the Kyoto Encyclopedia of Genes and Genomes (KEGG) database. Annotation genes: The total number of annotated genes. Unknown gene: The number of genes with unannotated functions.

Supplementary Table S4 Analysis results of transcription factors of *Lygus pratensis* in response to exogenous stress

Gene ID	Family	S-VS-R6 up/down	S-VS-R14 up/down	Description
Unigene0007479	SF-like	up	up	nuclear hormone receptor FTZ-F1 isoform X3
Unigene0043060	SF-like	up	up	nuclear hormone receptor FTZ-F1 beta
Unigene0011582	C_EBP	down	down	CCAAT/enhancer-binding protein beta-like
Unigene0019303	TF_bZIP	up	down	nuclear factor interleukin-3-regulated protein
Unigene0021221	TF_bZIP	down	down	basic leucine zipper transcriptional factor ATF-like 3
Unigene0051176	TF_bZIP	up	up	cyclic AMP-dependent transcription factor ATF-2 isoform X2
Unigene0045657	RXR-like	up	up	transcription factor HNF-4 homolog isoform X3
Unigene0007980	bHLH	up	up	upstream stimulatory factor 1
Unigene0015880	bHLH	up	up	protein atonal-like
Unigene0017761	bHLH	down	down	single-minded homolog 2 isoform X3
Unigene0029714	bHLH	down	down	transcription factor 21-like
Unigene0035330	bHLH	up	up	neurogenic differentiation factor 6-B-like
Unigene0043574	bHLH	down	down	helix-loop-helix protein delilah-like
Unigene0044744	bHLH	up	up	transcriptional regulator Myc-A-like
Unigene0057752	bHLH	down	down	protein dimmed-like
Unigene0059806	bHLH	up	up	circadian locomoter output cycles protein kaput
Unigene0060679	bHLH	up	up	protein daughterless isoform X2
Unigene0061585	bHLH	up	up	enhancer of split mgamma protein-like
Unigene0061663	bHLH	up	up	max-like protein X isoform X1
Unigene0000194	zf-C2H2	down	down	steroid hormone receptor ERR1
Unigene0005013	zf-C2H2	up	up	zinc finger protein 568-like
Unigene0005080	zf-C2H2	up	up	zinc finger protein OZF-like
Unigene0008103	zf-C2H2	up	up	zinc finger protein PLAG1-like
Unigene0008622	zf-C2H2	up	up	zinc finger protein 2 isoform X2
Unigene0011245	zf-C2H2	up	up	zinc finger protein 98
Unigene0011559	zf-C2H2	up	up	zinc finger protein GLI2-like
Unigene0011726	zf-C2H2	down	down	zinc finger protein 629-like
Unigene0012130	zf-C2H2	up	up	zinc finger protein Noc-like
Unigene0015377	zf-C2H2	up	up	zinc finger protein
Unigene0015937	zf-C2H2	up	up	zinc finger protein 629-like isoform X4
Unigene0018323	zf-C2H2	up	up	zinc finger protein 569-like
Unigene0030756	zf-C2H2	down	down	zinc finger protein 709-like
Unigene0041325	zf-C2H2	down	down	zinc finger protein GLI2 isoform X1
Unigene0043186	zf-C2H2	up	up	gastrula zinc finger protein XlCGF57.1-like
Unigene0043238	zf-C2H2	up	up	zinc finger protein 671-like

Unigene0043405	zf-C2H2	up	up	zinc finger protein 91-like
Unigene0043838	zf-C2H2	up	up	zinc finger protein 93-like
Unigene0044161	zf-C2H2	down	down	zinc finger protein Gfi-1b-like
Unigene0045242	zf-C2H2	up	up	zinc finger protein 708-like
Unigene0048751	zf-C2H2	up	up	zinc finger Y-chromosomal protein 1 isoform X1
Unigene0049326	zf-C2H2	up	up	zinc finger protein 62 homolog isoform X1
Unigene0049402	zf-C2H2	up	up	zinc finger protein SNAI2-like
Unigene0049418	zf-C2H2	down	down	zinc finger protein CKR1-like
Unigene0050556	zf-C2H2	up	up	zinc finger protein Xfin-like
Unigene0050851	zf-C2H2	up	up	zinc finger protein 782-like isoform X1
Unigene0051739	zf-C2H2	up	up	zinc finger protein 567-like isoform X2
Unigene0052797	zf-C2H2	down	down	zinc finger protein 287
Unigene0056021	zf-C2H2	up	up	zinc finger protein 600-like
Unigene0056222	zf-C2H2	up	up	zinc finger protein 367-like
Unigene0057426	zf-C2H2	up	up	zinc finger protein 70-like
Unigene0058080	zf-C2H2	up	up	zinc finger protein 236-like isoform X1
Unigene0058533	zf-C2H2	up	up	zinc finger protein 62 homolog isoform X1
Unigene0058899	zf-C2H2	up	up	gastrula zinc finger protein XlCGF49.1-like
Unigene0061020	zf-C2H2	up	up	zinc finger protein ush isoform X2
Unigene0061828	zf-C2H2	up	down	myeloid zinc finger 1-like isoform X2
Unigene0061941	zf-C2H2	up	up	zinc finger protein 91
Unigene0062520	zf-C2H2	up	up	zinc finger protein 341-like
Unigene0062773	zf-C2H2	up	up	zinc finger protein 91-like
Unigene0063267	zf-C2H2	up	up	zinc finger protein 569 isoform X1
Unigene0078695	zf-C2H2	down	down	zinc finger protein 184-like
Unigene0081968	zf-C2H2	up	up	zinc finger protein 664-like

Note: Gene ID: The unique identifier for each gene. Family: The family to which the transcription factor belongs (e.g., bHLH, zf-C2H2). S-VS-R6 up/down: The upregulation (up) or downregulation (down) of gene expression when comparing Sample S to Sample R6. S-VS-R14 up/down: The upregulation (up) or downregulation (down) of gene expression when comparing Sample S to Sample R14.

Supplementary Table S5 RT-qPCR Standard Curve Slopes and Amplification Efficiencies for Selected Genes

Gene ID	Gene Name	Slope	Amplification Efficiency (%)
Unigene0000972	Ada2b	-3.32	99.5
Unigene0002646	POU2F2	-3.28	100.2
Unigene0004283	His2B	-3.35	98.7%
Unigene0014063	ZNF776	-3.30	100.6%
Unigene0039154	DRGX	-3.33	99.3%
Unigene0030621	Gzf1	-3.29	100.1%
Unigene0041285	DRGX	-3.34	98.9%
Unigene0043936	Mecom	-3.31	100.4%
Unigene0057980	Twist2	-3.32	99.6%
Unigene0074021	ADA2	-3.30	100.5%

Note: Gene ID: A unique identifier assigned to each gene for differentiation based on the transcriptome database. Gene name: The name or symbol of the gene, facilitating reference in literature and databases.

Supplementary Table S6 RT-qPCR primers for validating transcriptome sequencing data

Gene ID	Gene Name	Primer Sequences	Product Size
Unigene0066231	Daam1	F：TCTCTGGAGGTTCGTCATCC	171
		R：TTCTCATCGGATCCTTGGAC	
Unigene0066420	ZNF678	F：GGCTGGGAAATCTGCAGTC	260
		R：TCTCGCCAGTGTGAATCC	
Unigene0000937	pol	F：TCTTTTCACCCGGCTCTTTA	150
		R：CCTCCACCGATTCTTTTTGA	
Unigene0033883	thap11	F：CCTCAGCCTGAAAACCAAAG	179
		R：GGTCTGAAACCAACCAGGAA	
Unigene0045937	Prpf40b	F：GGAGTCCTGAGTCTCGCAAC	150
		R：CAAGGGCAATTTGAAAGCAT	
Unigene0064136	ERCC6	F：GCTAGCTTTCACCGTTCGAC	133
		R：GCTTTTGGTTCGTTGTGGTT	
Unigene0000083	GEMIN8	F：GAAGATTGCCTCTTGCCATC	124
		R：ATGGGCCAGTATGGAGGATT	
Unigene0000194	Scrt1	F：CAAACCCACTCAGCTGACAA	144
		R：CCCTTTGCTGCTACTGAACC	
Unigene0000538	hsp70	F：AAATGTGGCCGTTCAAAGTC	168
		R：GCTTGTCGTTGGGAGTCATT	
Unigene0044998	Pka-R2	F：TTGAAGTAATCGCCCAGGTC	144
		R：CGTTAGACACGTACGCAGGA	
Genebank NO.	β-tubulin*	F：GCCGAATCCAACATGAACGA	159
ON798725.1		R：AATTGCAGACTTGGCGTTGT	

Note: Gene ID: A unique identifier assigned to each gene for differentiation based on the transcriptome database. Gene name: The name or symbol of the gene, facilitating reference in literature and databases. Primer sequences: The

sequences of primers used for RT-qPCR validation. "F" denotes the forward primer, and "R" denotes the reverse primer. Product size: The length of the RT-qPCR amplification product (in base pairs), used to confirm the correctness of the amplified fragment. *: The *β-tubulin* gene was identified and validated as the stably-expressed reference gene as the reference reported by Jia et al (2019).

Supplementary Table S7 Information of RT-qPCR primers for validation of transcription factor sequencing data

Gene ID	Gene Name	Primer Sequences	Product Size
Unigene0000972	Ada2b	F: TTCCACCGCTAGACATCA	145
		R: TCGTCATCAGCATTCATAGT	
Unigene0002646	POU2F2	F: AACCAGAAGACCTGAGTAAC	206
		R: CTGTACCATGAGCTGACTTA	
Unigene0004283	His2B	F: AAGTGGTTCAACGAGTCTAA	169
		R: TTACAGCCGAAGGACCTT	
Unigene0014063	ZNF776	F: GCTATGAAGAAGGCTGGAA	236
		R: ATTGTGGACCTCTTGTTGTA	
Unigene0039154	DRGX	F: GCATCACATCTACATACTCTG	206
		R: GTTCTGAGGTGGTACTTCA	
Unigene0030621	Gzf1	F: TTGGAGATCAGAAGCAGAAT	170
		R: TGCTCCTTCAGATGACTTAG	
Unigene0041285	DRGX	F: CGAAGCACGAGTTCAAGT	295
		R: TGGAGTTGGAGAAGTAATGG	
Unigene0043936	Mecom	F: CAGGACACAACGGAAGAA	383
		R: TGGTTCAGGAATGGATACG	
Unigene0057980	Twist2	F: CAGATTCGCAGCATCAAC	224
		R: TTCAGCCTCCTGTATGGT	
Unigene0074021	ADA2	F: ATAGATTGGACAAGCGATGA	348
		R: ATAGATTGGACAAGCGATGA	
Genebank NO.	β-tubulin*	F：GCCGAATCCAACATGAACGA	159
ON798725.1		R：AATTGCAGACTTGGCGTTGT	

Note: same as Supplementary Table 1.

*: The *β-tubulin* gene was identified and validated as the stably-expressed reference gene as the reference reported by Jia et al (2019).

Description: A functional description of the gene or the name of the homologous protein.

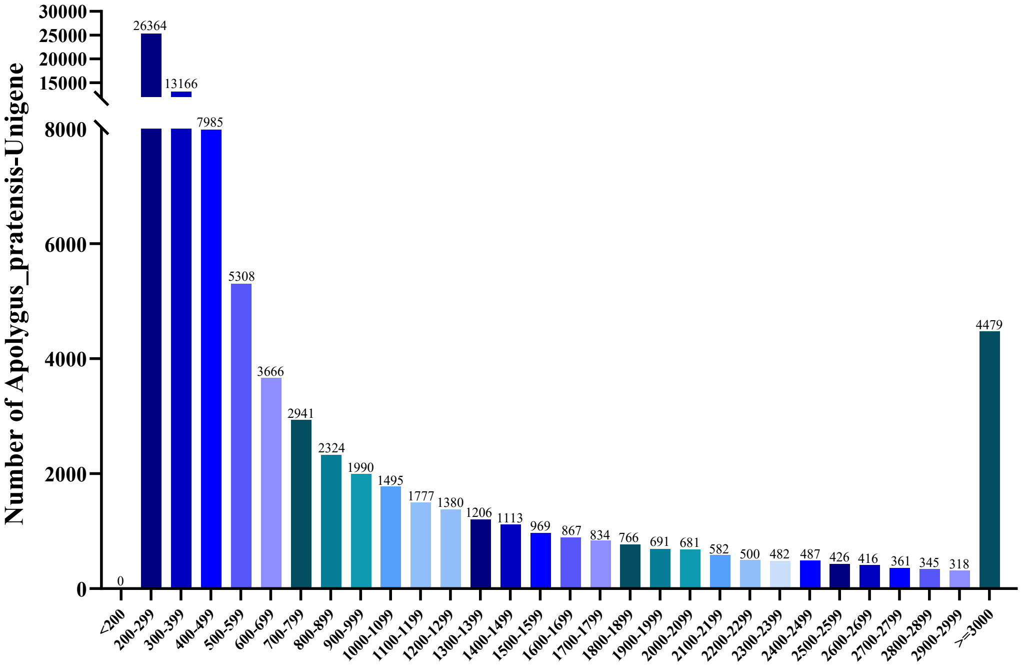

Supplementary Figure S1 Unigene length distribution of *Lygus pratensis*

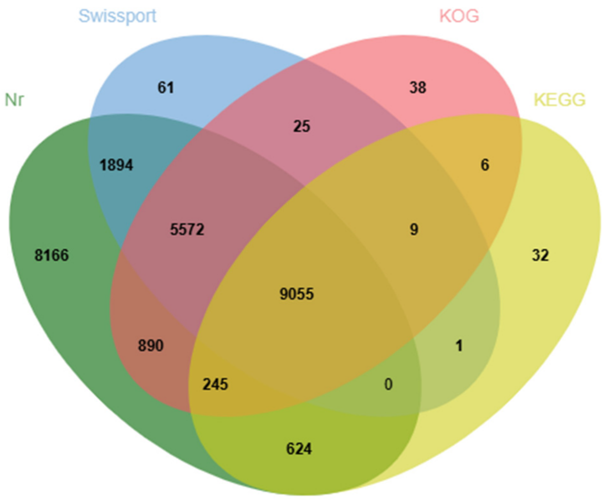

Supplementary Figure S2 Four databases annotation Venn diagram

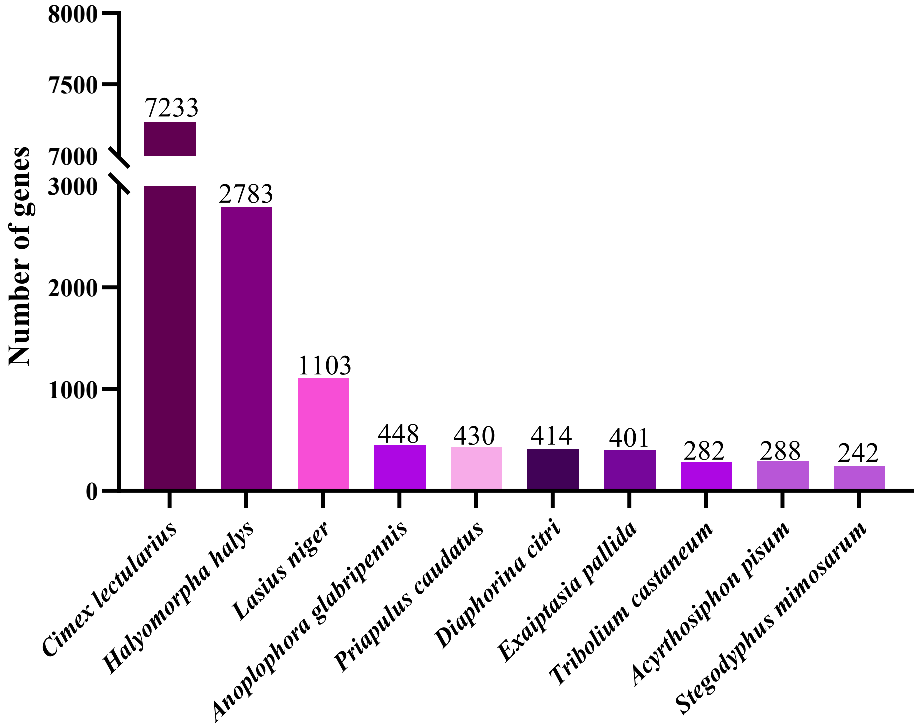

Supplementary Figure S3 Comparative distribution of homologous species of *Lygus pratensis* based on Nr annotation

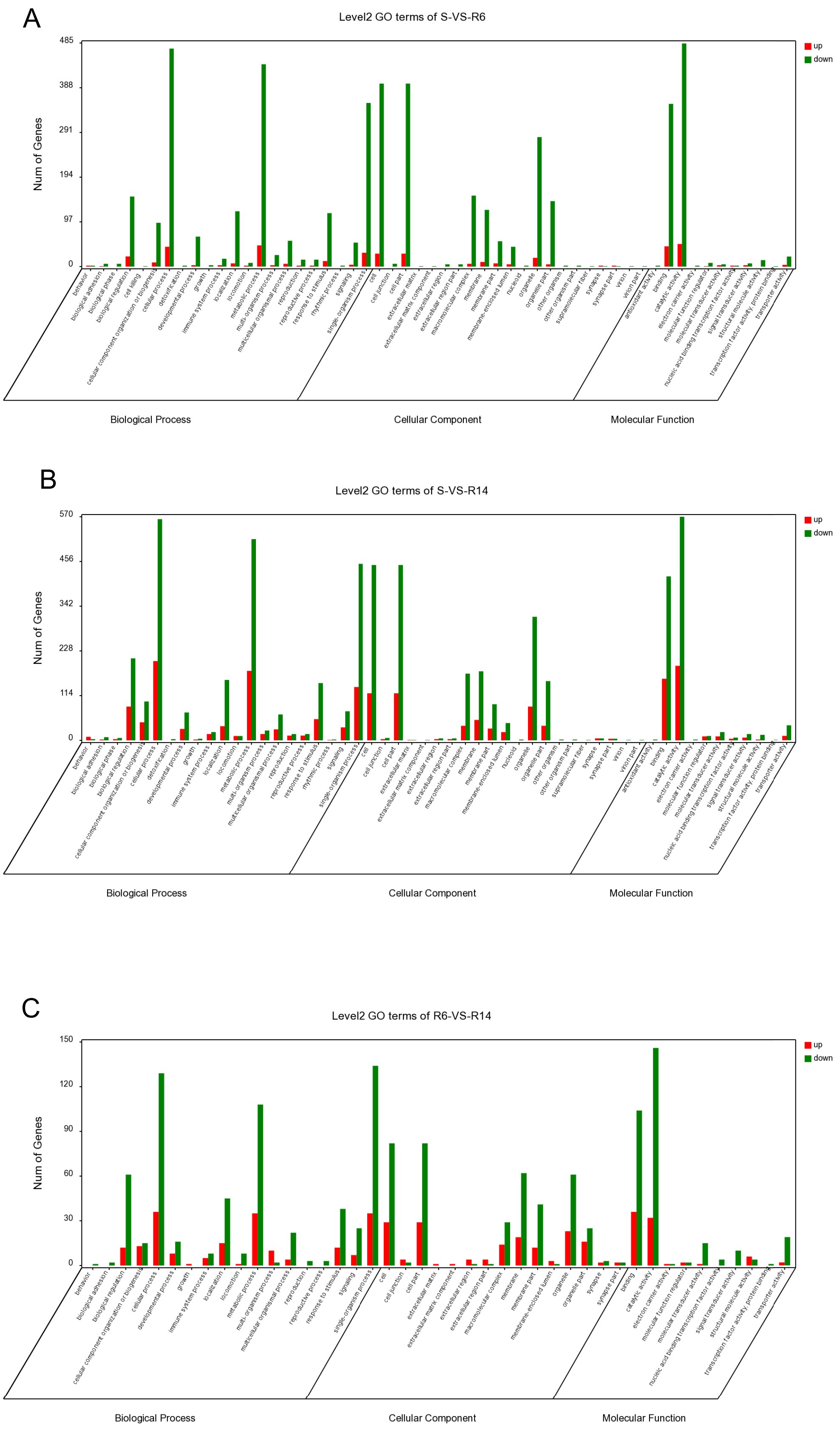

Supplementary Figure S4 Differential expression genes (DEGs) GO function annotation

A：S-VS-R6；B：S-VS-R14；C：R6-VS-R14

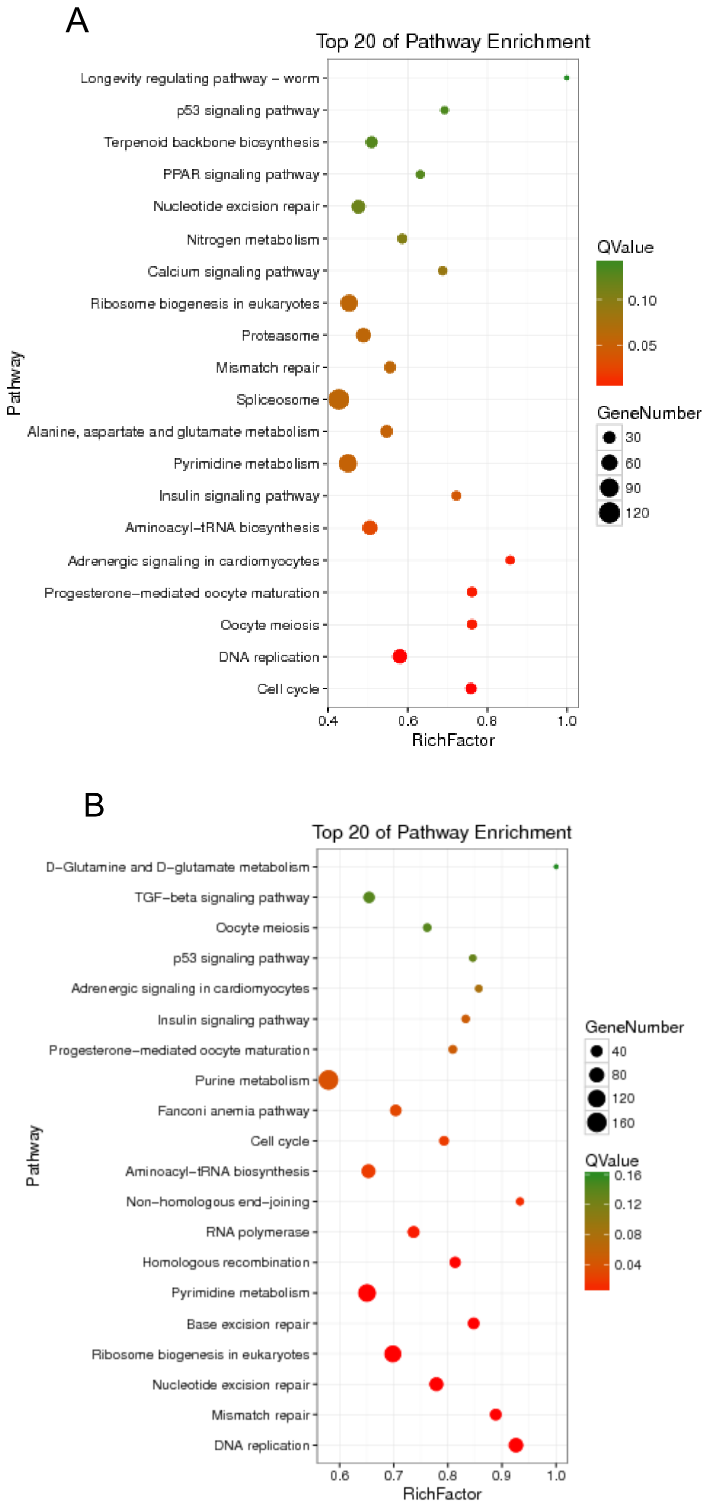
A
Top 20 of Pathway Enrichment
Longevity regulating pathway – worm
p53 signaling pathway
Terpenoid backbone biosynthesis
PPAR signaling pathway
Nucleotide excision repair
Nitrogen metabolism
Calcium signaling pathway
Ribosome biogenesis in eukaryotes
Proteasome
Mismatch repair
Spliceosome
Alanine, aspartate and glutamate metabolism
Pyrimidine metabolism
Insulin signaling pathway
Aminoacyl–tRNA biosynthesis
Adrenergic signaling in cardiomyocytes
Progesterone–mediated oocyte maturation
Oocyte meiosis
DNA replication
Cell cycle
Pathway
0.4 0.6 0.8 1.0
RichFactor
QValue
0.10
0.05
GeneNumber
30
60
90
120
B
Top 20 of Pathway Enrichment
D–Glutamine and D–glutamate metabolism
TGF–beta signaling pathway
Oocyte meiosis
p53 signaling pathway
Adrenergic signaling in cardiomyocytes
Insulin signaling pathway
Progesterone–mediated oocyte maturation
Purine metabolism
Fanconi anemia pathway
Cell cycle
Aminoacyl–tRNA biosynthesis
Non–homologous end–joining
RNA polymerase
Homologous recombination
Pyrimidine metabolism
Base excision repair
Ribosome biogenesis in eukaryotes
Nucleotide excision repair
Mismatch repair
DNA replication
Pathway
0.6 0.7 0.8 0.9 1.0
RichFactor
GeneNumber
40
80
120
160
QValue
0.16
0.12
0.08
0.04

C

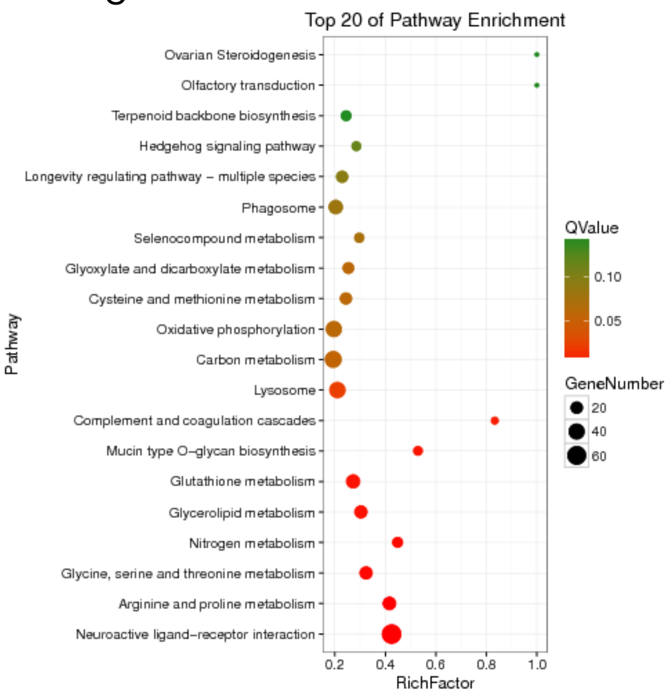

Supplementary Figure S5 Top 20 KEGG metabolic pathways enriched with differentially expressed genes (DEGs) A：S-VS-R6；B：S-VS-R14；C：R6-VS-R14

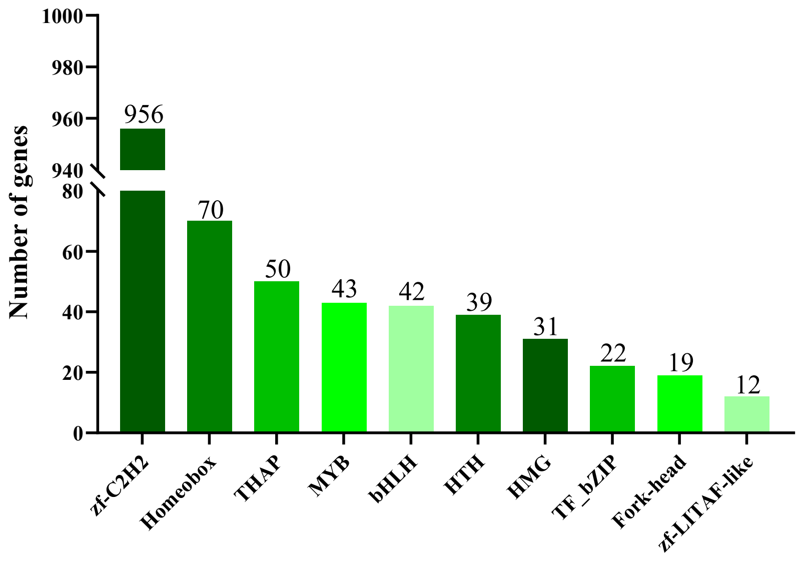

Supplementary Figure S6 Gene number of top 10 transcription factor family

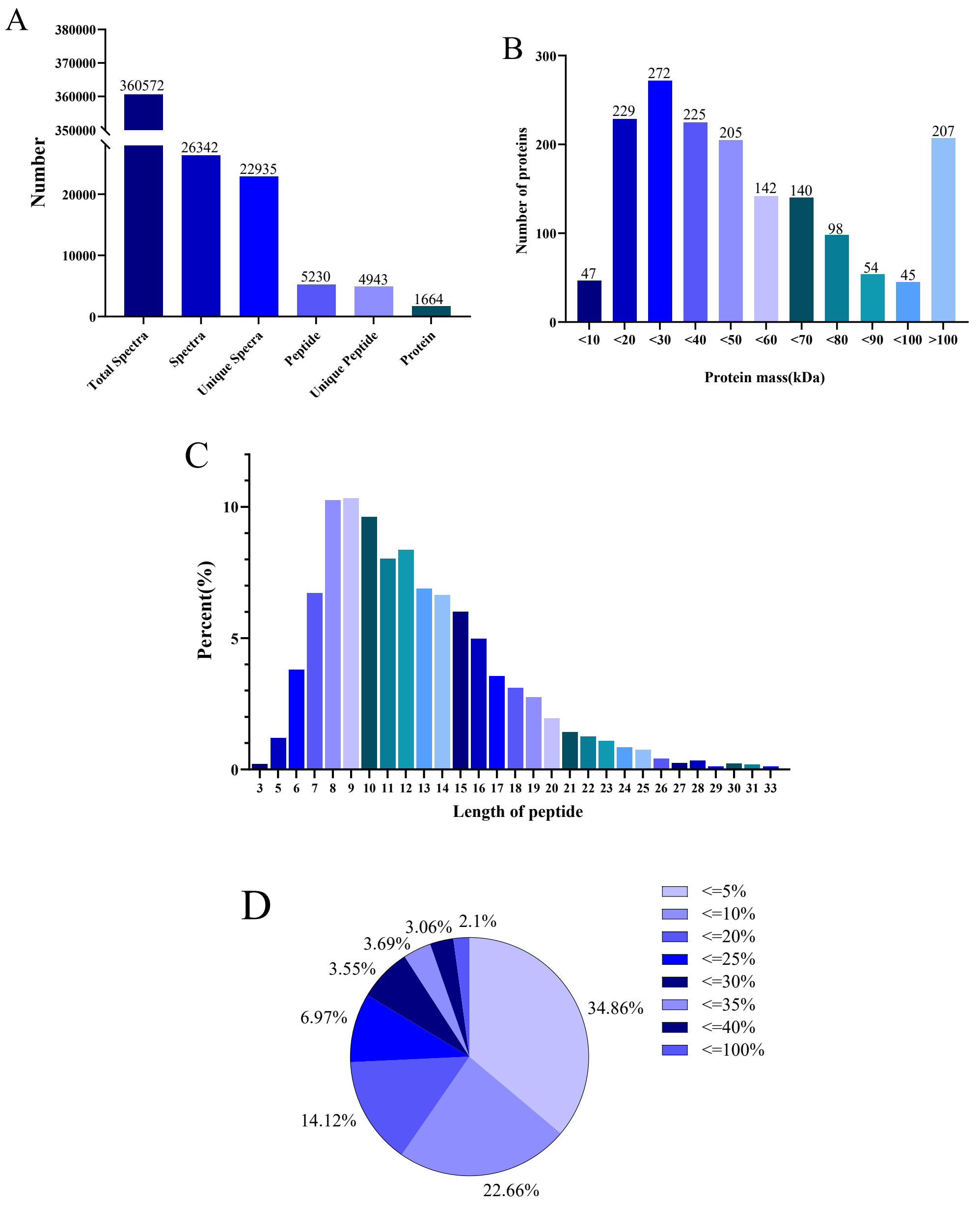

Supplementary Figure S7 Identification and analysis of proteins

A: Basic information of the identified protein; B: molecular weight distribution of the protein; C: peptide length distribution; D: peptide sequence coverage distribution map.

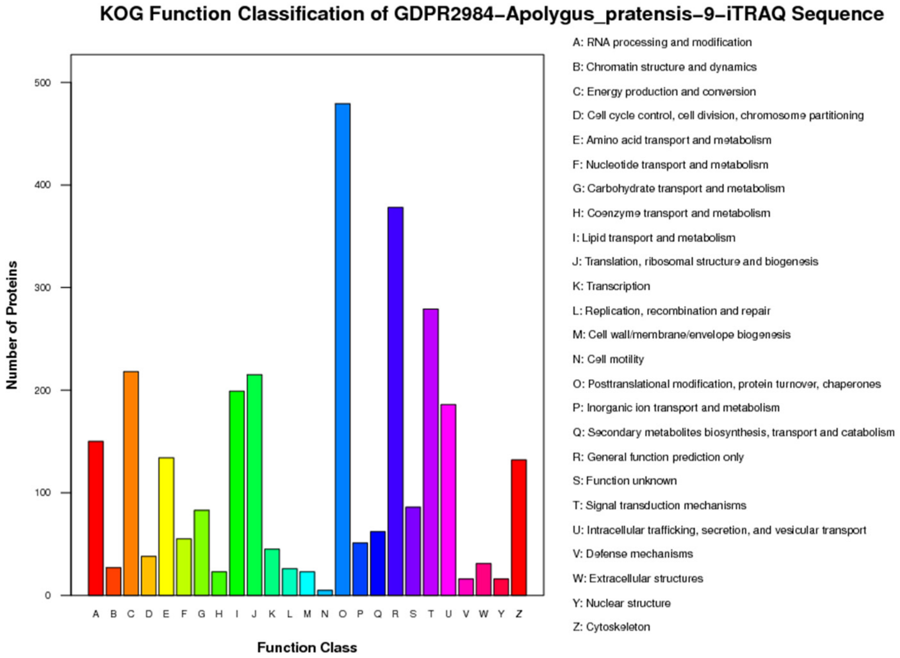

Supplementary Figure S8 KOG functional classification of differentially-expressed proteins

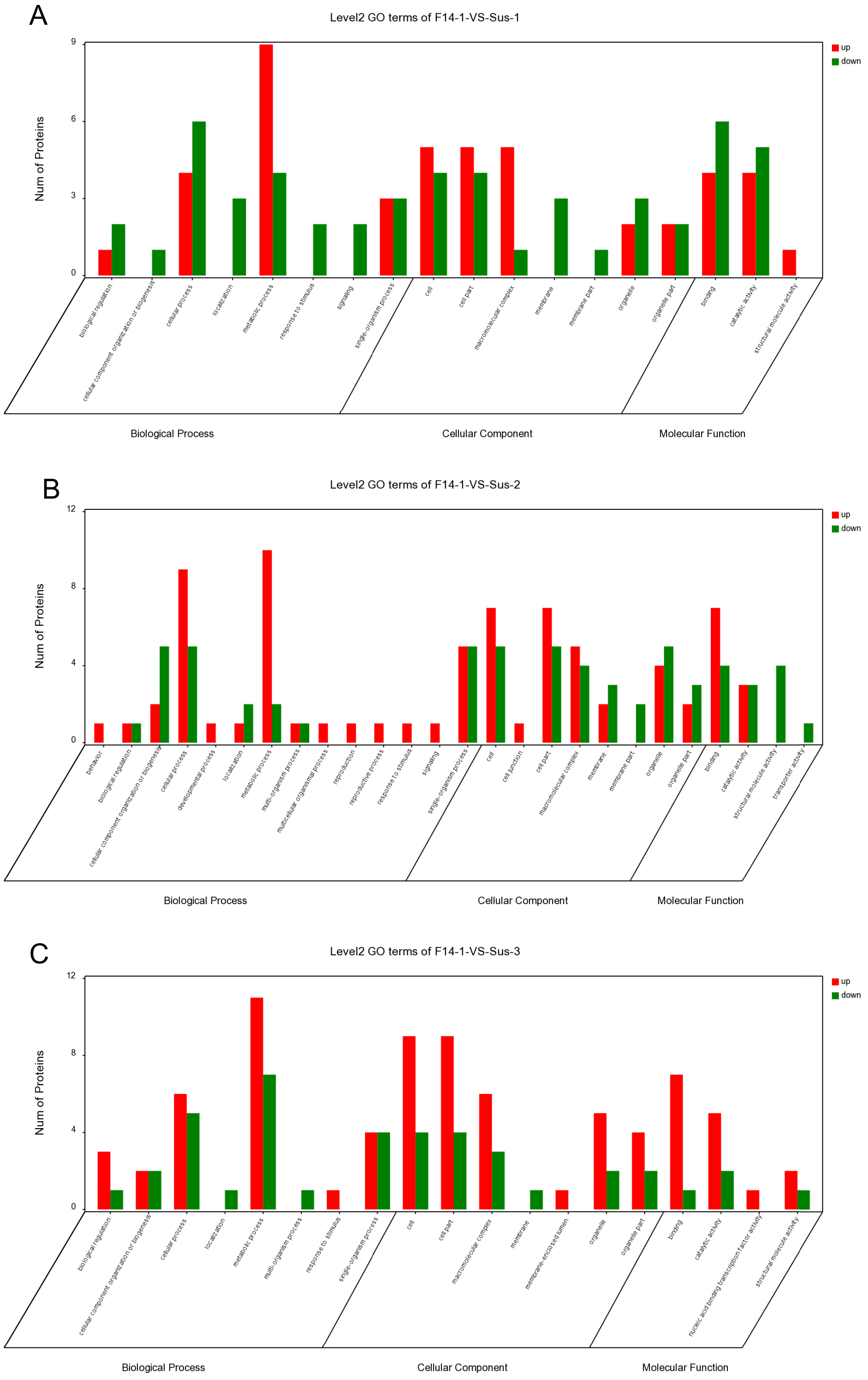

Supplementary Figure S9 GO enrichment analysis of differentially expressed proteins

A: F14-VS-Sus1; B: F14-VS- Sus2; C: F14-VS- Sus3.

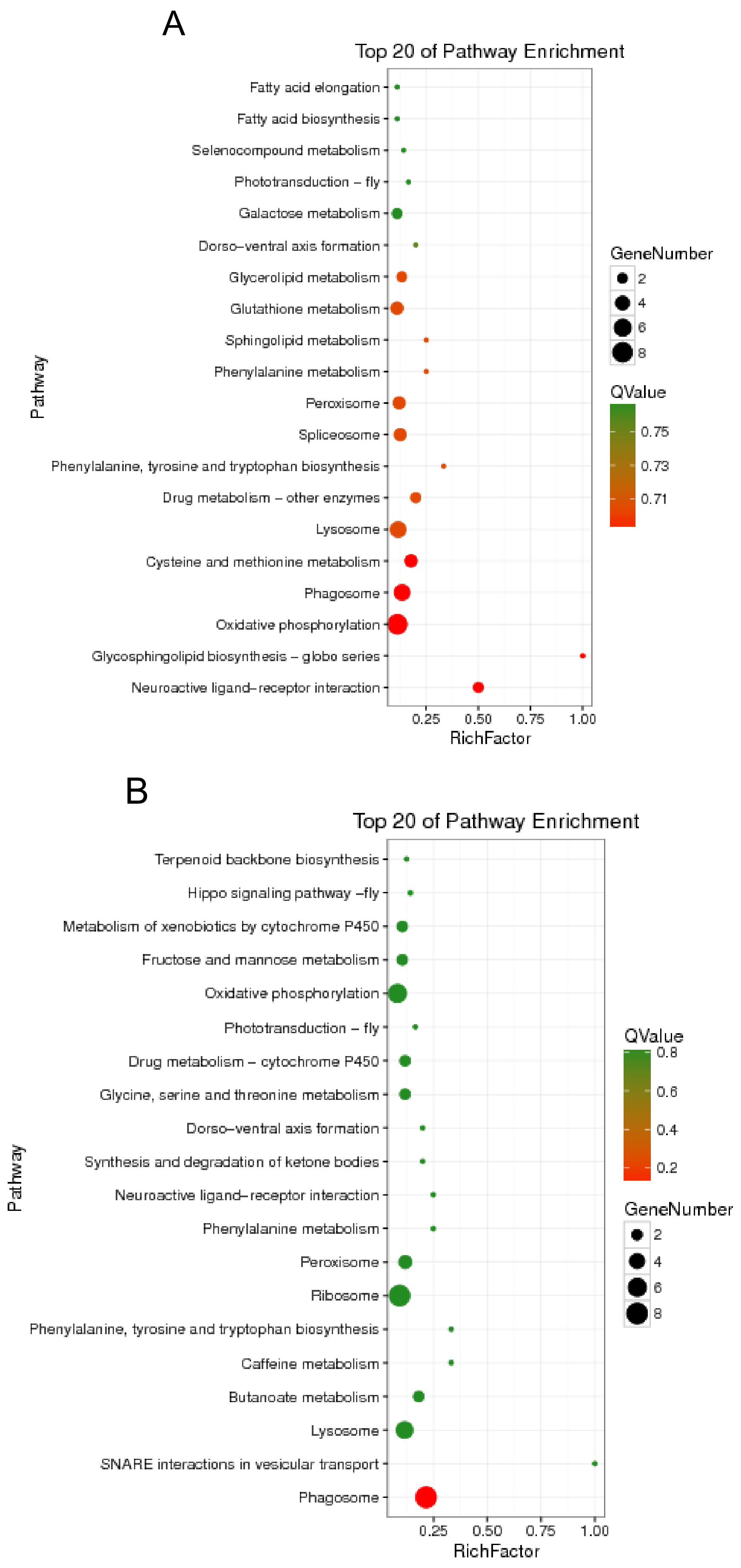

A
Top 20 of Pathway Enrichment
Fatty acid elongation
Fatty acid biosynthesis
Selenocompound metabolism
Phototransduction – fly
Galactose metabolism
Dorso–ventral axis formation
Glycerolipid metabolism
Glutathione metabolism
Sphingolipid metabolism
Phenylalanine metabolism
Peroxisome
Spliceosome
Phenylalanine, tyrosine and tryptophan biosynthesis
Drug metabolism – other enzymes
Lysosome
Cysteine and methionine metabolism
Phagosome
Oxidative phosphorylation
Glycosphingolipid biosynthesis – globo series
Neuroactive ligand–receptor interaction
Pathway
0.25 0.50 0.75 1.00
RichFactor
GeneNumber
2
4
6
8
QValue
0.75
0.73
0.71
B
Top 20 of Pathway Enrichment
Terpenoid backbone biosynthesis
Hippo signaling pathway –fly
Metabolism of xenobiotics by cytochrome P450
Fructose and mannose metabolism
Oxidative phosphorylation
Phototransduction – fly
Drug metabolism – cytochrome P450
Glycine, serine and threonine metabolism
Dorso–ventral axis formation
Synthesis and degradation of ketone bodies
Neuroactive ligand–receptor interaction
Phenylalanine metabolism
Peroxisome
Ribosome
Phenylalanine, tyrosine and tryptophan biosynthesis
Caffeine metabolism
Butanoate metabolism
Lysosome
SNARE interactions in vesicular transport
Phagosome
Pathway
0.25 0.50 0.75 1.00
RichFactor
QValue
0.8
0.6
0.4
0.2
GeneNumber
2
4
6
8

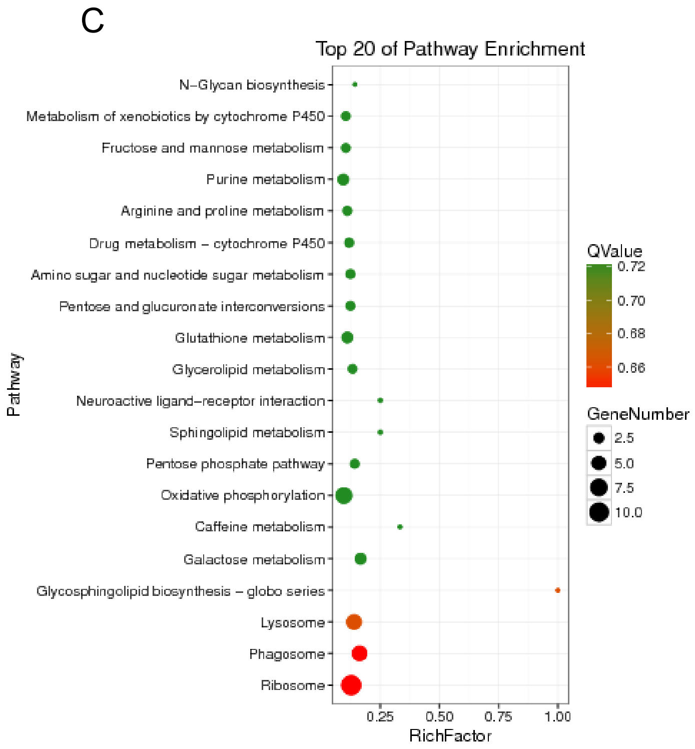

Supplementary Figure S10 The top 20 KEGG metabolic pathways

A: F14-VS-Sus1; B: F14-VS- Sus2; C: F14-VS- Sus3
